# Metabolomics and machine learning approaches for diagnostic and prognostic biomarkers screening in sepsis

**DOI:** 10.1186/s12871-023-02317-4

**Published:** 2023-11-09

**Authors:** Han She, Yuanlin Du, Yunxia Du, Lei Tan, Shunxin Yang, Xi Luo, Qinghui Li, Xinming Xiang, Haibin Lu, Yi Hu, Liangming Liu, Tao Li

**Affiliations:** 1 Department of Anesthesiology, Daping Hospital, Army Medical University, Chongqing, 400042 China; 2 State Key Laboratory of Trauma, Burns and Combined Injury, Shock and Transfusion Department, Daping Hospital, Army Medical University, Chongqing, 400042 China; 3 Department of Intensive Care Unit, Daping Hospital, Army Medical University, Chongqing, 400042 China

**Keywords:** Sepsis, Metabolomics, Biomarker, Machine learning, Phenylalanine metabolism

## Abstract

**Background:**

Sepsis is a life-threatening disease with a poor prognosis, and metabolic disorders play a crucial role in its development. This study aims to identify key metabolites that may be associated with the accurate diagnosis and prognosis of sepsis.

**Methods:**

Septic patients and healthy individuals were enrolled to investigate metabolic changes using non-targeted liquid chromatography-high-resolution mass spectrometry metabolomics. Machine learning algorithms were subsequently employed to identify key differentially expressed metabolites (DEMs). Prognostic-related DEMs were then identified using univariate and multivariate Cox regression analyses. The septic rat model was established to verify the effect of phenylalanine metabolism-related gene MAOA on survival and mean arterial pressure after sepsis.

**Results:**

A total of 532 DEMs were identified between healthy control and septic patients using metabolomics. The main pathways affected by these DEMs were amino acid biosynthesis, phenylalanine metabolism, tyrosine metabolism, glycine, serine and threonine metabolism, and arginine and proline metabolism. To identify sepsis diagnosis-related biomarkers, support vector machine (SVM) and random forest (RF) algorithms were employed, leading to the identification of four biomarkers. Additionally, analysis of transcriptome data from sepsis patients in the GEO database revealed a significant up-regulation of the phenylalanine metabolism-related gene MAOA in sepsis. Further investigation showed that inhibition of MAOA using the inhibitor RS-8359 reduced phenylalanine levels and improved mean arterial pressure and survival rate in septic rats. Finally, using univariate and multivariate cox regression analysis, six DEMs were identified as prognostic markers for sepsis.

**Conclusions:**

This study employed metabolomics and machine learning algorithms to identify differential metabolites that are associated with the diagnosis and prognosis of sepsis patients. Unraveling the relationship between metabolic characteristics and sepsis provides new insights into the underlying biological mechanisms, which could potentially assist in the diagnosis and treatment of sepsis.

**Trial registration:**

This human study was approved by the Ethics Committee of the Research Institute of Surgery (2021–179) and was registered by the Chinese Clinical Trial Registry (Date: 09/12/2021, ChiCTR2200055772).

**Supplementary Information:**

The online version contains supplementary material available at 10.1186/s12871-023-02317-4.

## Introduction

Sepsis is a significant public health concern characterized by a dysregulated host response to infection, resulting in life-threatening organ dysfunction [1]. The global burden of sepsis is increasing, with approximately 45 million new cases and 11 million deaths reported in 2017 [2]. Accurate definition, diagnosis, and early recognition of sepsis are crucial for improving treatment effects of patients. In addition to the early diagnostic symptoms and signs, the identification of available biomarkers is essential for monitoring and treating sepsis. Several molecules, including lactic acid, c-reactive protein (CRP), procalcitonin (PCT), and B-type natriuretic peptide (BNP), have been proposed as candidate sepsis biomarkers. However, the number of useful predictive biomarkers for assessing the severity and prognosis of sepsis in clinical practice remains limited [3–5]. Therefore, it is urgent to find specific biomarkers for sepsis.

Metabolomics is a novel approach developed following genomics, transcriptomics, proteomics and lipidomics [6, 7]. It involves the systematic identification and quantification of a wide range of small molecule metabolites in biological samples. Since the research focus is on endogenous molecule compounds at the end of metabolic pathways, this approach directly reflects the body's state, thereby offering a new means of identifying potential biomarkers [8]. Metabolomics has been extensively applied to investigate the pathologic mechanism and biomarker of sepsis. Mickiewicz et al. employed targeted metabolomics based on nuclear magnetic resonance (NMR) to identify 186 metabolites in the serum of patients in the Intensive Care Unit, suggesting that metabolomics holds promise for predicting mortality in septic shock [9]. Another study found that two metabolites could distinguish severe sepsis from systemic inflammatory response syndrome [10]. Liquid chromatography and tandem mass spectrometry (LC–MS/MS) combines high performance liquid chromatography with electrospray ionization mass spectrometry (ESI–MS) metabolomics technology to achieve comprehensive detection of different types of metabolites in samples [11]. In comparison to targeted metabolomics using NMR, LC–MS/MS offers the advantages of heightened sensitivity and a broader dynamic range [12].

Machine learning is a key area within the field of artificial intelligence [13, 14], representing an algorithmic framework that enables intelligent processing of data. Through feature extraction, machine learning continually enhances its performance by automatically learning internal data patterns. The main algorithms include artificial neural network (ANN), support vector machine (SVM), random forest (RF), and decision tree (DT) [15, 16]. Utilizing machine learning opens up new possibilities for enhancing diagnostic efficiency and achieving more objective and personalized patient assessments, deviating from traditional diagnostic and treatment methods [17]. Despite its increasing prominence in the diagnostic field, it is rarely used to identify potential diagnostic and prognostic goals for sepsis.

The present study employed liquid chromatography quadrupole time of flight mass spectrometry (LC-QTOF/MS) to analyze the serum metabolites of sepsis patients. Machine learning techniques were then utilized to identify potential biomarkers for the diagnosis and prognosis of sepsis.

## Materials and methods

### Ethical review

This human study was approved by the Ethics Committee of the Research Institute of Surgery (2021–179) and was registered by the Chinese Clinical Trial Registry (Date: 09/12/2021, ChiCTR2200055772). All the participants provided written informed consent before inclusion in this study. All experimental procedures were approved by the Animal Ethics Committee of Army Medical University, China, and were performed in accordance with the National Institutes of Health “Guidelines for the Care and Use of Laboratory Animals”. The study was carried out in compliance with the ARRIVE guidelines. All methods are reported in accordance with ARRIVE guidelines (https://arriveguidelines.org) for the reporting of animal experiments.

### Study design and population recruitment

From December 2021 to April 2022, a total of 30 septic patients were recruited from the Intensive Care Unit (ICU) of Daping Hospital, Army Medical University, based on the Sepsis-3 criteria for sepsis and septic shock. Additionally, 15 age-matched healthy volunteers were enrolled from the State Key Laboratory of Trauma, Burns, and Combined Injury. The inclusion and exclusion criteria for septic patients are presented in Supplementary Table 1. Healthy participants also follow the similar inclusion and exclusion criteria. Blood samples were collected from all septic patients within 24 h of admission, while blood samples from healthy volunteers were obtained during enrollment.

### Blood sample collection and metabolites extraction

A 10 mL peripheral venous blood sample was collected from all participants in both groups. Subsequently, the samples were centrifuged at 3000 rpm for 10 min, and the resulting supernatant was aliquoted into 1.5 mL centrifuge tubes and stored at -80 °C. For analysis, 100 μL of each sample was transferred to an EP tube. To this, 400 μL of extract solution (methanol containing isotopically-labelled internal standard mixture) was added, followed by 30 s of vortexing, 10 min of sonication in an ice-water bath, and 1 h of incubation at -40 °C to precipitate proteins. The sample was then centrifuged at 12,000 rpm (RCF = 13,800(× g), *R* = 8.6 cm) for 15 min at 4 °C. The resulting supernatant was transferred to a fresh glass vial for analysis. A quality control (QC) sample was prepared by combining equal aliquots of the supernatants from all the individual samples.

### LC–MS/MS analysis

LC–MS/MS analyses were conducted using a UHPLC system (Vanquish, Thermo Fisher Scientific) coupled to an Orbitrap Exploris 120 mass spectrometer (Orbitrap MS, Thermo) with a UPLC HSS T3 column (2.1 mm × 100 mm, 1.8 μm). The mobile phase consisted of 5 mmol/L ammonium acetate and 5 mmol/L acetic acid in water (A) and acetonitrile (B). The auto-sampler temperature was maintained at 4 °C, and the injection volume was 2 μL. The Orbitrap Exploris 120 mass spectrometer was selected for its capability to acquire MS/MS spectra in information-dependent acquisition (IDA) mode, controlled by Xcalibur software (Thermo). In this mode, the acquisition software continuously evaluates the full scan MS spectrum. The ESI source conditions were set as follows: sheath gas flow rate at 50 Arb, aux gas flow rate at 15 Arb, capillary temperature at 320 °C, full MS resolution at 60,000, MS/MS resolution at 15,000, collision energy at 10/30/60 in NCE mode, and spray voltage at 3.8 kV (positive) or -3.4 kV (negative), respectively.

### Data preprocessing and annotation

The raw data were converted to the mzXML format using ProteoWizard and processed with an in-house program developed in R. This program utilized XCMS for peak detection, extraction, alignment, and integration. Metabolite annotation was performed using an in-house MS2 database called BiotreeDB, with a cutoff set at 0.3 for annotation.

### Identification of differentially expressed metabolites via RF and SVM Algorithms

In this study, various machine learning algorithms were employed to identify differentially expressed metabolites (DEMs). The random forest (RF) algorithm was utilized to determine the optimum DEMs between the sepsis and healthy control groups. Using the "e1071" R package, the support vector machine (SVM) generated eigenvectors were eliminated to extract the optimal variables for identifying diagnostic DEMs in sepsis. Additionally, a SVM classifier was constructed with tenfold cross-validation. Finally, a Venn diagram was employed to identify the shared DEMs.

### Dataset collection

A total of 18 genes related to phenylalanine metabolism were obtained from the Molecular Signatures Database (MSigDB) (https://www.gsea-msigdb.org/gsea/) for further investigation. The mRNA expression matrix was downloaded from the Gene Expression Omnibus (GEO) dataset (https://www.ncbi.nlm.nih.gov/geo/). In this study, the GSE57065 dataset was utilized for screening differentially expressed genes (DEGs). The R package "SVA" was employed to normalize the gene expression matrices and eliminate batch effects. Perl scripts were used to convert the dataset probes into corresponding gene symbols.

### Sepsis model establishment

The ethics and protocols for the animal experiments were approved by the La-boratory Animal Welfare and Ethics Committee of the Army Medical University (Approval No. AMUWEC20224867). Adult male and female Sprague–Dawley rats weighing between 200-220 g were bred in a facility with filtered positive-pressure ventilation, under a 12:12-h dark/light cycle, with ad libitum access to food and water. The rats were randomly assigned to three groups: Control (*n* = 30), Sepsis (*n* = 30), and RS8359-treated Sepsis (*n* = 30). The RS8359 compound (HY-14260, Med-ChemExpress) was administered at a dosage of 5 mg/kg, dissolved in ddH2O, through the tail vein 30 min prior to inducing sepsis in the RS8359-treated group. The Sepsis group received an equivalent volume of ddH2O. The rats were anesthetized with sodium pentobarbital (45 mg/kg, i.p.) for the surgical procedure. Cecal ligation and puncture (CLP) were conducted to create the sepsis model following established protocols [8]. Myocardial tissues were harvested at 12 h after CLP and stored at − 80 °C until further processing.

### Statistical analysis

All statistical analyses were conducted using R software (version 4.1.2, http://www.r-project.org). The DEGs were identified using the "Limma" package with a significance threshold of *P* < 0.05. Receiver operating characteristic (ROC) analysis was performed, and the area under the curve (AUC) was calculated to assess the predictive performance of the classifier.

## Results

### Clinical characteristics of the studied population

Our study included 30 septic patients and 15 healthy controls. The patients in the sepsis group had a mean age of 60.1 ± 14.5, while the control group had a mean age of 50.4 ± 17.2 (*p* > 0.05). There were no significant differences in gender and BMI among the two groups (*p* > 0.05). However, we observed significant differences in temperature (*p* = 0.01), heart rate (*p* = 0.001), and white blood cell count (*p* = 0.003) when comparing septic patients to the healthy controls (Table 1).

**Table 1:** Baseline characteristics of the septic patients and healthy controls in this study

**Characteristics**		**Septic patients**	**Healthy controls**	***p*-Value**
		**(*n* = 30)**	**(*n* = 15)**	
Age, yrs	Mean	60.1	50.4	0.053
	SD	14.5	17.2	
Gender, n (%)	Female	10 (33.3)	6 (40.0)	0.660
	Male	20 (66.7)	9 (60.0)	
BMI, kg/m^2^	Mean	24.8	25.1	0.779
	SD	3.2	4.3	
Mean arterial Pressure, mmHg	Mean	86.6	88.3	0.632
	SD	11.8	9.2	
Temperature, ℃	Mean	36.9	36.5	0.01
	SD	0.5	0.3	
White blood cell count, × 10^9^/L	Mean	16.9	6.6	0.003
	SD	12.2	3.1	
Heart rate/min	Mean	94.9	79.1	0.001
	SD	15.3	8.3	

### Metabolic reprogramming related differentially expressed metabolites participated in the occurrence of sepsis

**Fig. 1:**
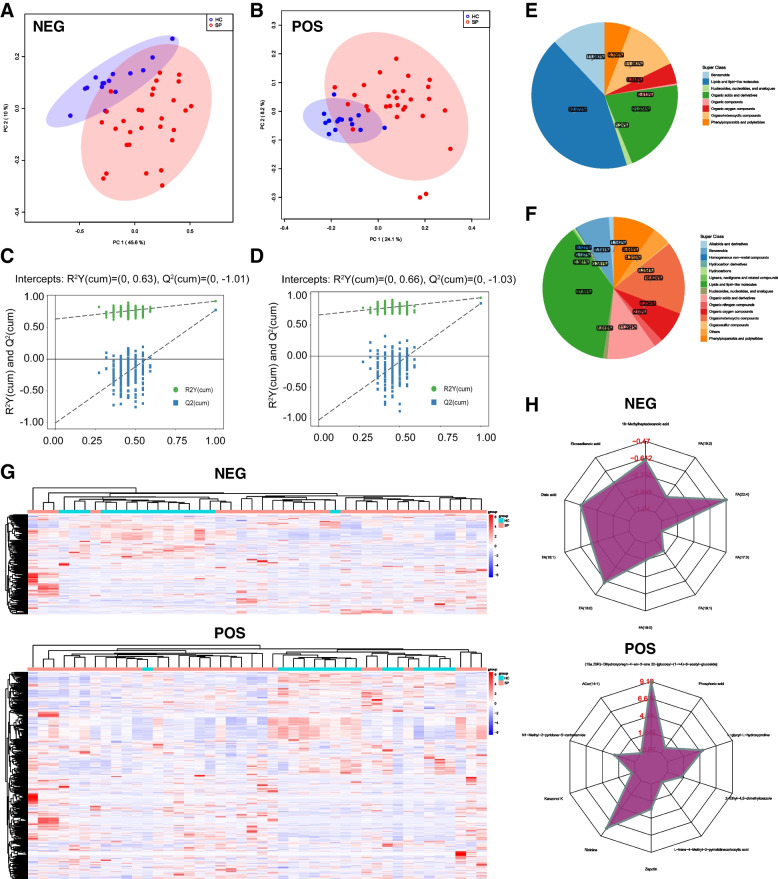
Identification of differentially expressed metabolites (DEMs) between control and sepsis. **A**,** B** Principal component analysis (PCA) scores plot for metabolomics analysis in the sepsis and control groups. PC1 and PC2 in the figure represent the scores of the first and second principal components respectively. Each scatter represents a sample. The red circle represents septic patients, and the blue circle represents the healthy controls. **C, D** OPLS-DA replacement test of NEG and POS. The abscissa represents the displacement retention degree of the displacement test, the ordinate represents the value of R^2^Y or Q^2^, the green dot represents the R^2^Y value obtained from the displacement test, the blue square dot represents the Q^2^ value obtained from the displacement test, and the two dotted lines represent the regression lines of R^2^Y and Q^2^ respectively. Pie charts of super class of DEMs in NEG **E** and POS (**F**) models. (**G**) Heat maps and (**H**) Radar plots analyzed by TBtools showing the significantly changed metabolites in sepsis and control

The ionization source of Orbitrap is electrospray ionization, including positive and negative ion modes (POS and NEG, respectively), which can increase the metabolite coverage and improve the detection effect. Subsequent data analysis involved separate analyses of the two groups. Principal component analysis (PCA) revealed significant differences in metabolites between sepsis patients (SP) and healthy controls (HC) (Fig. 1A, B). Further assessment using Orthogonal Partial Least Squares-Discriminant Analysis (OPLS-DA) demonstrated the robustness of the original model without any overfitting observed (Fig. 1C, D). Metabolomics analysis detected a total of 532 differentially expressed metabolites (DEMs) (VIP > 1, *P* < 0.05) between the sepsis and control groups, with 327 identified in the positive ion mode and 205 in the negative ion mode (Fig. 1G). The pie plots based on super class classification revealed that the DEMs primarily belonged to Lipids and lipid-like molecules (37.69% in POS and 42.975% in NEG) and Organic acids and derivatives (11.294% in POS and 21.074% in NEG) (Fig. 1 E, F). Identified through radar plots, the top 10 DEMs with the highest fold change between sepsis patients and healthy controls in POS were (15a,20R)-Dihydroxypregn-4-en-3-one 20-[glucosyl-(1- > 4)-6-acetyl-glucoside], Acar (14:1), N1-Methyl-2-pyridone-5-carboxamide, Kanzonol K, Ricinine, Zapotin, L-trans-4-Methyl-2-pyrrolidinecarboxylic acid, 2-Ethyl-4,5-dimethyloxazole, L-glycyl-L-hydroxyproline, and Phosphoric acid. Meanwhile, in NEG, the top 10 DEMs were 16-Methylheptadecanoic acid, Eicosadienoic acid, Oleic acid, FA (18:1), FA (18:0), FA (19:0), FA (19:1), FA (17:0), FA (22:4), and FA (19:2) (Fig. 1H).

**Fig. 2:**
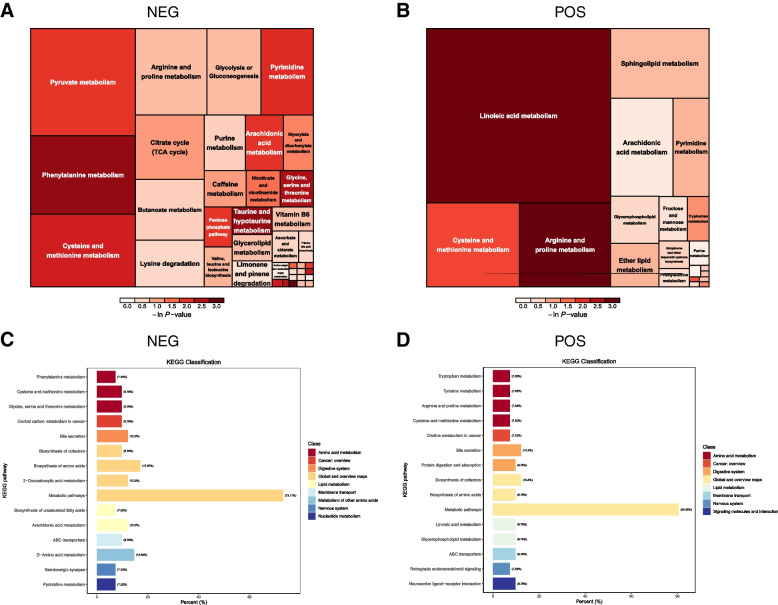
Correlation and function enrichment analysis of DEMs. Pathway analysis of DEMs in (**A**) NEG and (**B**) POS models. Each square in the rectangular tree represents a metabolic pathway. The size of the square represents the impact degree of the pathway, and the color represents the *P* value. **C**, **D** Kyoto Encyclopedia of Genes and Genomes (KEGG) enrichment analysis of DEMs in NEG and POS. The vertical axis represents the names of the enriched KEGG metabolic pathways, and the horizontal axis represents the percentage of annotated metabolites in each pathway relative to the total number of metabolites

Figure 2A and B illustrate that the differentially expressed metabolites (DEMs) pathways primarily involved Phenylalanine metabolism, Pyruvate metabolism, and Cysteine and methionine metabolism in the NEG model. On the other hand, in the POS model, the DEMs were enriched in Linoleic acid metabolism and Arginine and proline metabolism. To further assess the functional implications of these metabolites, we conducted Kyoto Encyclopedia of Genes and Genomes (KEGG) pathway enrichment analysis. In the NEG model, the most significant KEGG pathways included biosynthesis of amino acids, phenylalanine metabolism, cysteine and methionine metabolism, as well as glycine, serine, and threonine metabolism. Meanwhile, in the POS model, KEGG analysis revealed significant enrichment in pathways related to tyrosine metabolism, arginine and proline metabolism, and linoleic acid metabolism (Fig. 2C, D). These findings indicate a close association between sepsis progression and metabolic reprogramming.

### Identification of key DEMs in sepsis via various machine learning algorithms

**Fig. 3:**
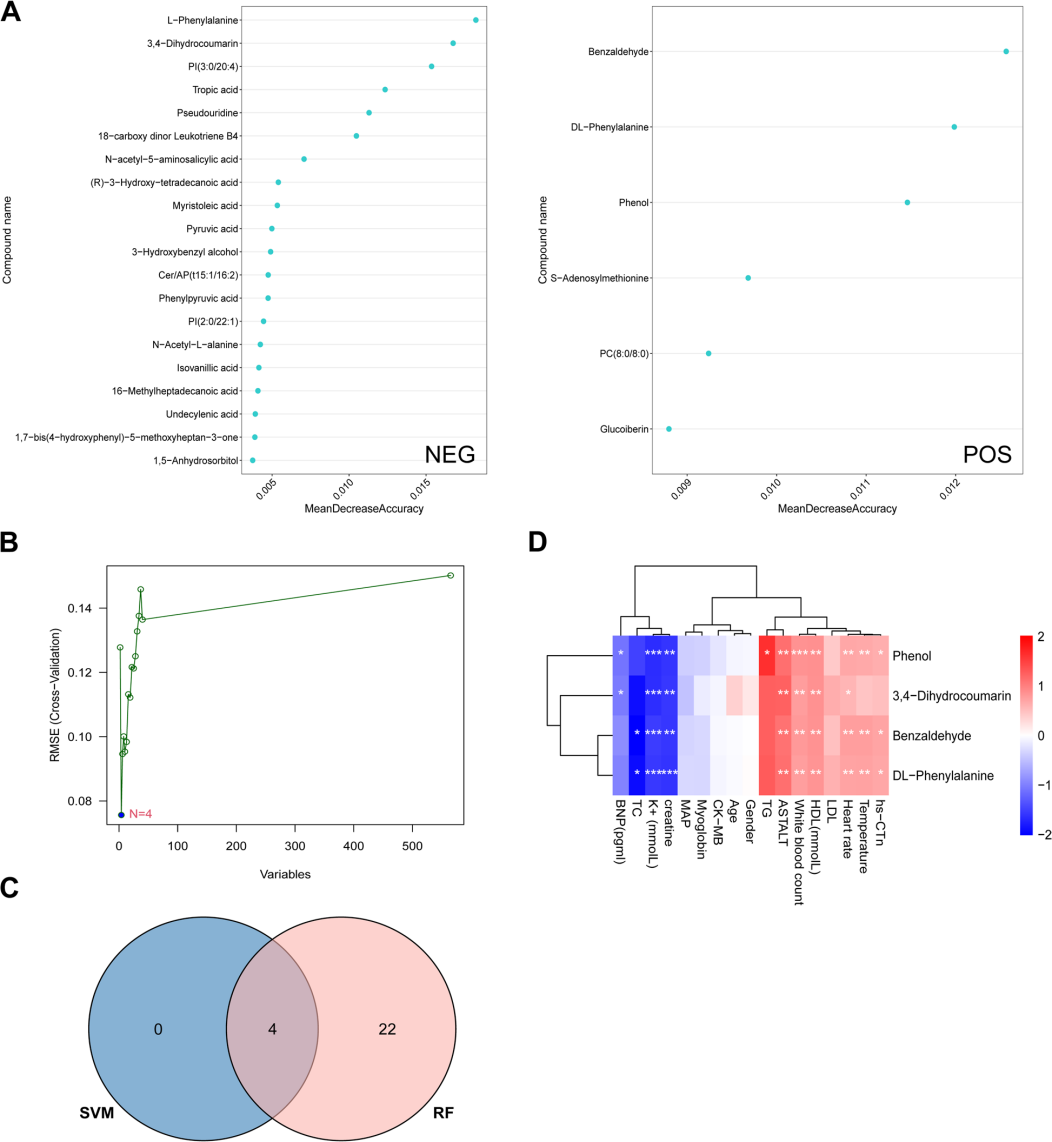
Screening of DEMs via the comprehensive strategy. **A** DEMs screened by random forest (RF) algorithm. **B** Support vector machine (SVM) was applied for DEMs. **C** VENN diagram of hub DEMs. **D** Person’s correlation analysis between hub DEMs and routine detection indexes. *P < 0.05, **P < 0.01, ***P < 0.001

We constructed a Random Forest (RF) model using minimum error regression trees for screening differentially expressed metabolites (DEMs) (Fig. 3A). Through this approach, we identified 26 DEMs in both the NEG and POS models. Additionally, we established a support vector machine (SVM) for DEM selection (Fig. 3B). By comparing the results from RF and SVM, we determined 4 key DEMs (3,4-Dihydrocoumarin, Phenol, Benzaldehyde, and DL-Phenylalanine) based on the intersection of these two methods (Fig. 3C). Furthermore, we conducted Pearson's correlation analysis to examine the relationship between these key DEMs and conventional indicators, which is represented in the correlation thermogram (Fig. 3D). The analysis revealed positive correlations between the 4 DEMs and high sensitivity cardiac troponin (hs-CTn), temperature, heart rate, high density lipoprotein (HDL), white blood count, aspartate aminotransferase/alanine aminotransferase (AST/ALT), and triglyceride (TG). Conversely, a negative correlation was observed between the 4 DEMs and creatine, potassium (K^+^), total cholesterol (TC), and B-type natriuretic peptide (BNP).

### The 4 DEMs were diagnostic biomarkers of sepsis

**Fig. 4:**
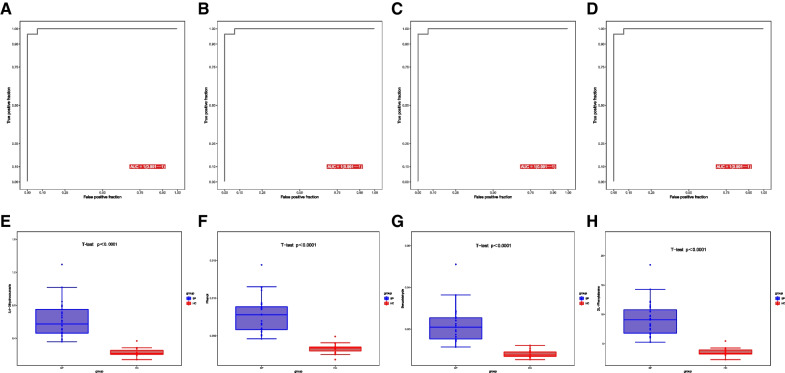
Receiver operating characteristic curve (ROC) analysis of the 4 DEMs. ROC curve of (**A**) 3,4-Dihydrocoumarin, (**B**) Phenol, (**C**) Benzaladehyde, and (**D**) DL-Phenylalanine. The expression of (**E**) 3,4-Dihydrocoumarin, (**F**) Phenol, (**G**) Benzaladehyde, and (H) DL-Phenylalanine between sepsis and control groups

We further screened metabolites by ROC analysis and calculated the area under the curve (AUC). The results showed that the AUC of the 4 key DEMs analyzed by machine learning were both 1.0, which showed an excellent prediction ability for sepsis (Fig. 4A-D). The expressions of the 4 key DEMs (3,4-Dihydrocoumarin, Phenol, Benzaladehyde, and DL-Phenylalanine) were higher in the sepsis group than in the control group (Fig. 4E-H).

### The phenylalanine metabolism-related key gene MAOA played important role in sepsis

**Fig. 5:**
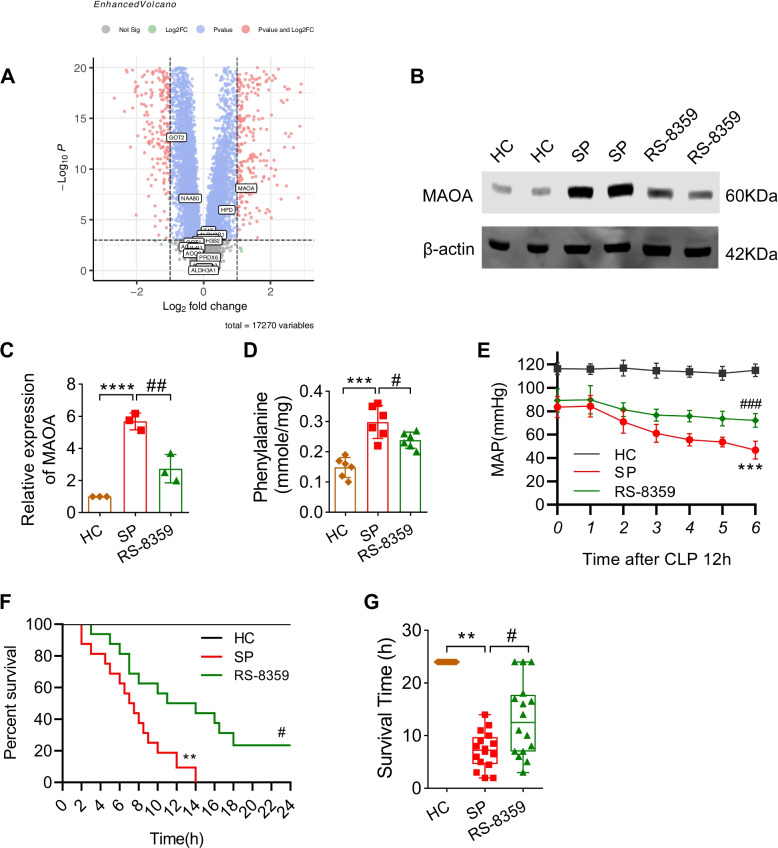
The role of MAOA in sepsis rats. **A** Volcano plot of differentially-expressed genes (DEGs) of GSE57065. Statistically significant DEGs were identified as those with a student’s t-test (|logFC|> 1, *P* < 0.001). **B** Representative Western blotting images and (**C**) quantification of Western blotting results of MAOA between the control group, the sepsis group and the RS-8359 group in heart tissues (*n* = 3 independent experiments). ***P < 0.001 vs control, ###*P* < 0.001 vs sepsis. **D** The level of phenylalanine in heart tissues detected by the phenylalanine assay kit (ab241000, Abcam) (*n* = 6 each group). ***P < 0.001 vs control, #*P* < 0.05 vs sepsis. (**E**) MAP within 6 h detecting starting at 12 h after CLP between the control group, the sepsis group and the RS-8359 group (*n* = 8 each group). ***P < 0.001 vs control, ###*P* < 0.001 vs sepsis. **F** The survival rate of rats between the control group, the sepsis group and the RS-8359 group (*n* = 16 each group). **P < 0.01 vs control, #*P* < 0.05 vs sepsis. **G** The survival time of rats in between the control group, the sepsis group and the RS-8359 group. **P < 0.01 vs control, #*P* < 0.05 vs sepsis

Due to the enrichment analysis of differential metabolites and machine learning screening showing the significant role of phenylalanine in the progression of sepsis, we further investigated the role and regulatory mechanism of phenylalanine metabolism in sepsis. We analyzed transcriptome data from the GEO database. This dataset included 25 blood samples from healthy controls and 82 blood samples from septic patients. We identified differentially expressed genes (DEGs) between the sepsis and healthy control groups using a threshold of |log2FC|> 1 and *p* < 0.001. As depicted in Fig. 5A (volcano plot), Monoamine Oxidase A (MAOA), the sole phenylalanine metabolism-related gene, exhibited a significant increase post-sepsis. To demonstrate the impact of MAOA in sepsis, we employed a sepsis model in rats using CLP (cecal ligation and puncture) and observed the effect of MAOA inhibition through its antagonist, RS-8359. RS-8359 was administered through the tail vein at a dosage of 5 mg/kg during sepsis induction. Firstly, we confirmed the inhibitory effect of RS-8359 on MAOA in myocardial tissue via Western blot analysis (Fig. 5B, C). Subsequently, our study revealed that mean arterial pressure (MAP) decreased after CLP, while administration of RS-8359 increased the MAP compared to the sepsis group (Fig. 5D). None of the septic rats survived beyond 24 h, with an average survival time of 7.25 ± 3.49 h. Conversely, in the RS-8359 group, 25% (4/16) of the rats survived for 24 h, with an average survival time of 13.16 ± 7.07 h, which was significantly longer than the sepsis group (Fig. 5E, F).

#### DEMs could indicate the prognosis for sepsis

**Fig. 6:**
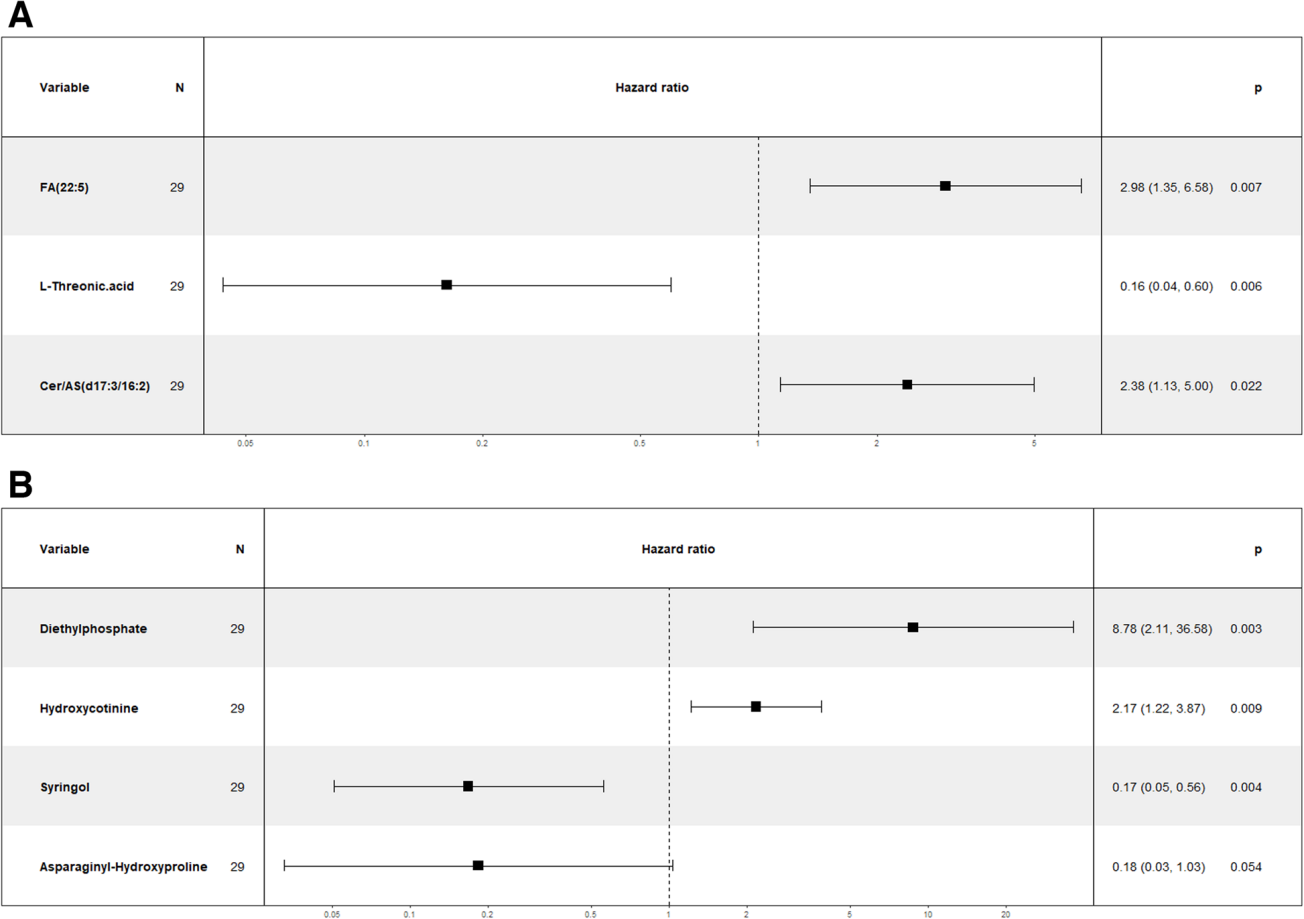
Prognostic analysis of DEMs in sepsis. Multivariate Cox regression analysis of DEMs between septic patients and healthy controls in (**A**) NEG and (**B**) POS models

In the study, we followed up with patients for 28 days from the diagnosis of sepsis in the ICU, the 28 days mortality of septic patients was 36.7% (11/30), and the ICU mortality was 13.3% (4/30). Univariate Cox regression and multivariate Cox regression analysis were used to identify the prognostic related DEMs. After observing the survival outcome of septic patients within 28 days, DEMs associated with survival were identified by univariate Cox regression analysis in NEG and POS (Tables 2 and 3). Subsequently, a total 6 prognostic related DEMs, including FA (22:5) (HR = 2.98, *p* = 0.007), L-Threonic (HR = 0.16, *p* = 0.006), Cer/AS(d17:3/16:2) (HR = 2.38, *p* = 0.022), Diethylphosphate (HR = 8.78, *p* = 0.003), Hydroxycotinine (HR = 2.17, *p* = 0.009) and Syringol (HR = 0.17, *P* = 0.004) were identified by multivariate Cox regression analysis (Fig. 6A, B).

**Table 2:** Univariate Cox regression analysis of DEMs in NEG

variable	coef	HR (95%CI)	wald.*p* value	logtest.*p* value	scoretest.*p* value
1,5-Anhydrosorbitol	0.6573	1.93 (1.083–3.438)	0.02574	0.04006	0.02025
Cer/AS (d17:3/16:2)	0.7347	2.085 (1.086–4.004)	0.02732	0.02391	0.02255
FA (22:5)	0.6023	1.826 (1.068–3.124)	0.0279	0.03729	0.02161
L-Threonic.acid	-1.112	0.3288 (0.1178–0.9178)	0.03369	0.008543	0.01793
Syringic.acid	0.85	2.34 (1.053–5.201)	0.03701	0.01919	0.02848
LPI (18:2)	0.6436	1.903 (1.012–3.581)	0.04591	0.0495	0.03913
Pseudouridine	-0.986	0.3731 (0.1401–0.9932)	0.04842	0.01554	0.02889

**Table 3:** Univariate Cox regression analysis of DEMs in POS

variable	coef	HR.95.CI	wald.*p* value	logtest.*p* value	scoretest.*p* value
Hydroxycotinine	0.6298	1.877 (1.197–2.945)	0.00612	0.02619	0.0001932
N-Arachidonoyl.GABA	-0.7594	0.4679 (0.2616–0.8372)	0.0105	0.01245	0.009885
3-Methylpyrrolo[1,2-a]pyrazine	-0.6216	0.5371 (0.3162–0.9122)	0.02144	0.03489	0.01837
Diethylphosphate	0.8103	2.249 (1.101–4.593)	0.02616	0.02566	0.02738
Syringol	-0.707	0.4931 (0.2583–0.9414)	0.0321	0.02855	0.02854
PE(3:0/22:5)	0.6309	1.879 (1.028–3.437)	0.04054	0.0434	0.03291
Isoferulic.acid.3-O-glucuronide	-0.7971	0.4506 (0.2075–0.9785)	0.04391	0.02584	0.03046
Asparaginyl-Hydroxyproline	-1.096	0.3342 (0.1125–0.9926)	0.04845	0.01329	0.03163

## Discussion

In this study, metabolomics analysis identified a total of 532 differentially expressed metabolites (DEMs). These DEMs were primarily involved in various metabolic pathways, including amino acid biosynthesis, phenylalanine metabolism, tyrosine metabolism, glycine, serine, and threonine metabolism, as well as arginine and proline metabolism. Machine learning algorithms SVM and RF were utilized to identify four metabolic biomarkers (3,4-Dihydrocoumarin, Phenol, Benzaladehyde, and DL-phenylalanine) that are associated with the diagnosis of sepsis. Additionally, an analysis of transcriptome data from sepsis patients in the GEO database revealed a significant up-regulation of the phenylalanine-metabolism-related gene MAOA in the sepsis group. Inhibition of MAOA with the inhibitor RS-8359 resulted in improved mean arterial pressure and survival rate in septic rats. Finally, univariate and multivariate Cox regression analysis identified six DEMs (FA (22:5), L-Thyronic, Cer/AS (d17:3/16:2), Diethylphophase, Hydroxyco-tinine, and Syringol) that are associated with the prognosis of sepsis.

Machine learning algorithms have played a crucial role in the mining of metabolomic data [18]. A lot of new biomarkers can be found by analyzing metabolomics based on machine learning. Fortino et al. [19] utilized comprehensive transcriptome analysis coupled with machine learning to identify 89 biomarkers and decipher disease-related characteristic genes. In another study, Bi-farin et al. [20] employed machine learning algorithms to analyze urine metabolomics of renal cell carcinoma (RCC) patients and successfully identified seven metabolites for the diagnosis of RCC, achieving an impressive AUC of up to 0.98. Similarly, in a study involving serum samples from 109 gout patients, 102 asymptomatic hyperuricemia patients, and 119 normal uric acid control groups, three machine learning algorithms (random forest, support vector machine, and logistic regression) were employed, leading to the identification of 13 metabolites as potential biomarkers to distinguish hyperuricemia, gout, and normal uric acid conditions [21]. Given the substantial amount of metabolomics information in sepsis, the use of intelligent algorithms to mine variables becomes crucial. In this particular study, the SVM and RF algorithms were employed to screen for four distinctive DEMs in the serum of sepsis patients. Subsequently, the diagnostic efficacy of these four metabolites was evaluated using ROC curve analysis, ultimately identifying them as potential new markers for sepsis diagnosis.

Previous studies have shown that bacterial infection can lead to an imbalance of amino acid metabolism [22]. In sepsis patients, several metabolic characteristics have been observed, including hypermetabolism, catabolism, negative nitrogen balance, muscle and visceral protein decomposition for energy, liver uptake of amino acids for gluconeogenesis, and significant metabolic changes in various amino acids [23]. Amino acid metabolism disorder has been found to play a vital role in sepsis progression. Chen et al. conducted a non-targeted metabolomics analysis on serum samples from sepsis patients and identified extensive amino acid metabolism abnormalities. Notably, phenylalanine and histidine metabolism exhibited the most significant alterations in sepsis [24]. Aromatic amino acids (phenylalanine) can compete with branched-chain amino acids to penetrate the blood–brain barrier. The increase of aromatic amino acids causes the formation of pseudo-neurotransmitters, thus inhibiting the central nervous system [25]. Another study also indicated the association between phenylalanine metabolism and sepsis-related acute renal injury [26]. Amino acids like arginine and glycine possess antioxidant and immunomodulatory effects [27]. Glycine, in particular, can enhance myocardial function and diminish the production of free radicals, thereby mitigating endotoxin-induced myocardial damage [28]. Furthermore, by acting on the glycine receptor (GlyR) on cell membranes, glycine can prevent the excessive activation of Kupffer cells and play a significant role in liver protection [29]. Thus, glycine serves as a crucial metabolic regulator in human cells. Citrulline also plays a vital role in cell metabolism and organ function regulation [30]. This study not only observed abnormal amino acid metabolism, including phenyl-alanine metabolism, tyrosine metabolism, glycine, serine, and threonine metabolism, and arginine and proline metabolism in sepsis patients, but also examined the expression of genes related to phenylalanine metabolism in the sepsis patient transcriptome and the protective effect of MAOA inhibitors on sepsis in rats.

This study utilized metabolomics, machine learning algorithms, and prognosis analysis to screen potential metabolic biomarkers associated with the diagnosis and prognosis of sepsis. Moreover, it specifically examined the alterations in phenylalanine metabolism following sepsis and subsequently analyzed the regulatory mechanism of MAOA. However, since this study is considered exploratory and based on a small sample size of very new biomarkers, we opted to use completely healthy individuals as the control group. This decision could potentially lead to an overestimation of the sensitivity and specificity of the biomarkers. In future large sample studies, we will select case-controls as the control group to authenticate the diagnostic efficacy of these biomarkers.

## Conclusions

The current study utilized metabolomics and machine learning algorithms to identify distinct metabolites associated with the diagnosis and prognosis of sepsis patients. Investigating the association between metabolic characteristics and sepsis offers novel insights into the underlying biological mechanisms, which may potentially enhance the diagnosis and treatment of sepsis.

### Supplementary Information

**Additional file 1: Supplementary Table 1.** The inclusion and exclusion criteria of septic patients.

**Additional file 2.**

## Data Availability

The datasets analyzed in this study are available from the corresponding authors upon reasonable request. Additionally, the datasets presented in this study can be found in an online repository, specifically at https://www.ncbi.nlm.nih.gov/geo/ with the accession number GSE57065.
