# Supplementary material for: Metabolomics and machine learning approaches for diagnostic and prognostic biomarkers screening in sepsis
Source: BMC Anesthesiol. 2023 Nov 9;23:367. doi: 10.1186/s12871-023-02317-4 (PMC10634148; doi:10.1186/s12871-023-02317-4)
Supplement: Supplementary file 1 — Additional file 1: Supplementary Table 1. The inclusion and exclusion criteria of septic patients. [file 12871_2023_2317_MOESM1_ESM.docx]

**Supplementary Table 1. The inclusion and exclusion criteria of septic patients.**

| Inclusion criteria | Diagnosed as sepsis |
| --- | --- |
|  | The patient informed consent and participated voluntarily |
|  | 18-80 years old (including 80 years old) |
|  | Register general medical conditions |
| Exclusion criteria | Age>80 years or ≤ 18 years |
|  | Previous history of chronic heart disease, liver and kidney diseases |
|  | Pregnant or lactating women |
|  | Hyperlipidemia, diabetes, or other metabolic diseases |
|  | Complicated with cardiovascular, liver, kidney and hematopoietic system and other serious primary diseases |
|  | Combined with psychiatric diseases |
|  | Long-term use of sedative drugs or alcohol |
|  | Complicated with tumor or immune deficiency and other diseases that have a greater impact on immunity |
|  | Patient or family refusal to be involved in the study |
