# Supplementary figures and images for: Metabolomics and machine learning approaches for diagnostic and prognostic biomarkers screening in sepsis
Source: BMC Anesthesiol. 2023 Nov 9;23:367. doi: 10.1186/s12871-023-02317-4 (PMC10634148; doi:10.1186/s12871-023-02317-4)

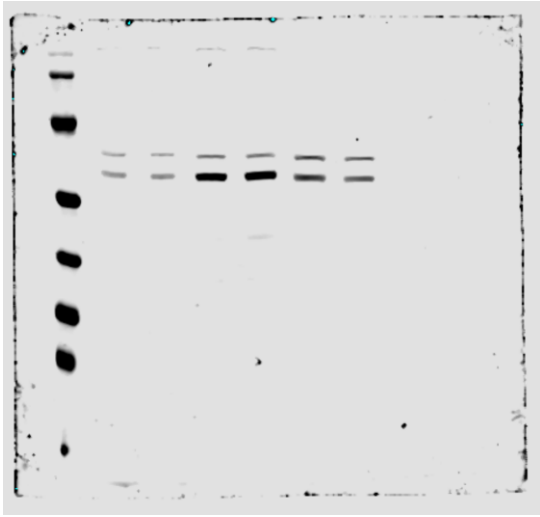

MAOA  
60KDa

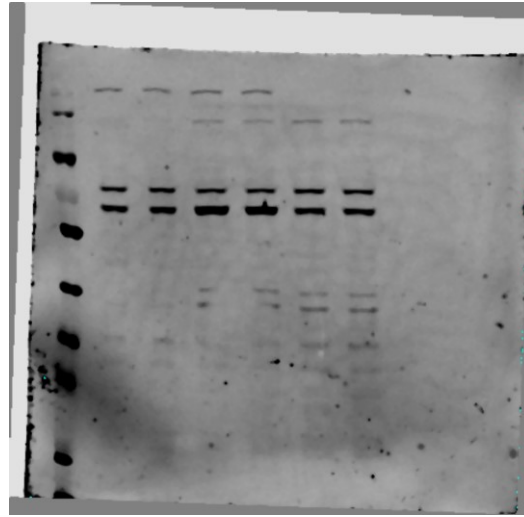

MAOA  
60KDa

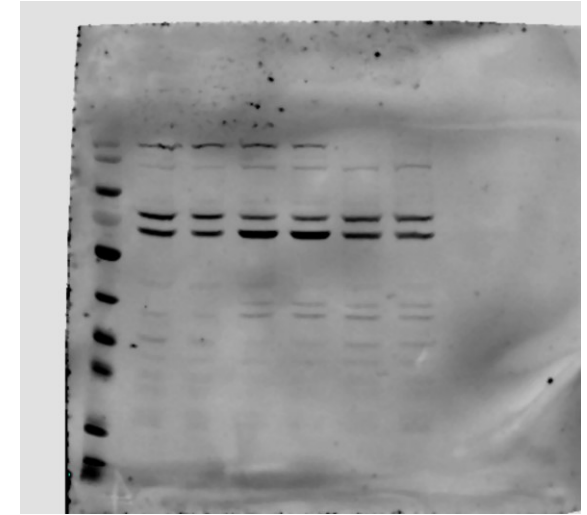

MAOA  
60KDa

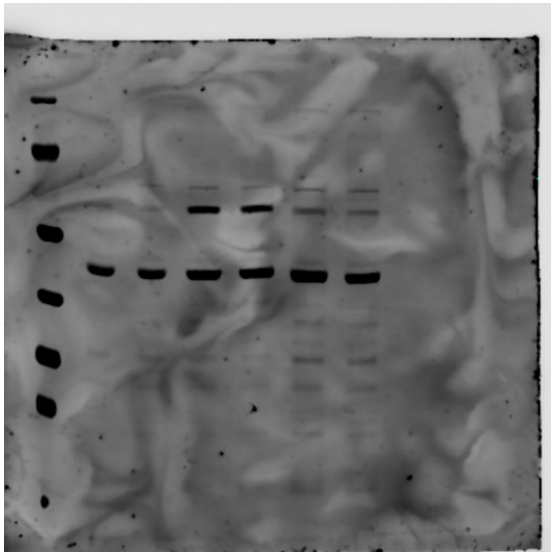

MAOA  
60KDa  
 $\beta$ -actin  
42KDa

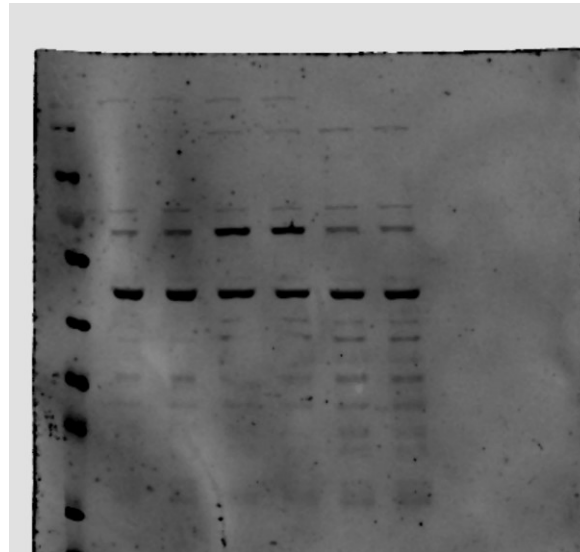

MAOA  
60KDa  
 $\beta$ -actin  
42KDa

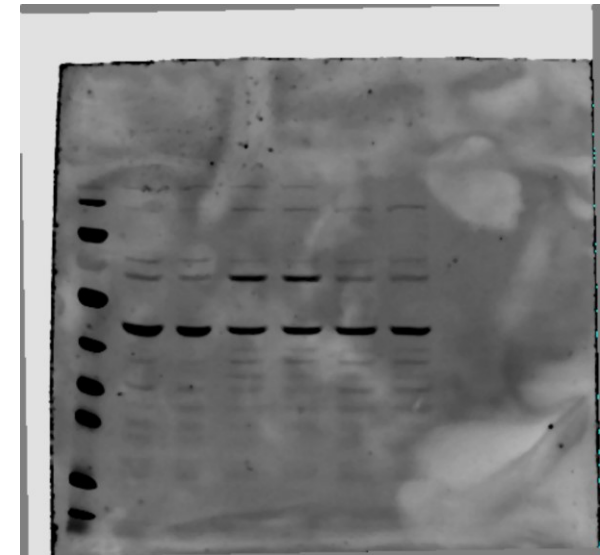

MAOA  
60KDa  
 $\beta$ -actin  
42KDa

Supplement: Supplementary file 2 — Additional file 2. [file 12871_2023_2317_MOESM2_ESM.pdf]
